# Identification of E-cadherin signature motifs functioning as cleavage sites for *Helicobacter pylori* HtrA

**DOI:** 10.1038/srep23264

**Published:** 2016-03-17

**Authors:** Thomas P. Schmidt, Anna M. Perna, Tim Fugmann, Manja Böhm, Jan Hiss, Sarah Haller, Camilla Götz, Nicole Tegtmeyer, Benjamin Hoy, Tilman T. Rau, Dario Neri, Steffen Backert, Gisbert Schneider, Silja Wessler

**Affiliations:** 1 Cancer Cluster Salzburg, Department of Molecular Biology, University of Salzburg, Austria; 2 Department of Chemistry and Applied Biosciences, Swiss Federal Institute of Technology (ETH), Zurich, Switzerland; 3 Philochem AG, Otelfingen, Switzerland; 4 Division of Microbiology, University of Erlangen-Nuremberg, Germany; 5 Institute of Pathology, University of Erlangen-Nuremberg, Germany; 6 Institute of Pathology, University of Bern, Bern, Switzerland

## Abstract

The cell adhesion protein and tumour suppressor E-cadherin exhibits important functions in the prevention of gastric cancer. As a class-I carcinogen, *Helicobacter pylori* (*H. pylori*) has developed a unique strategy to interfere with E-cadherin functions. In previous studies, we have demonstrated that *H. pylori* secretes the protease high temperature requirement A (HtrA) which cleaves off the E-cadherin ectodomain (NTF) on epithelial cells. This opens cell-to-cell junctions, allowing bacterial transmigration across the polarised epithelium. Here, we investigated the molecular mechanism of the HtrA-E-cadherin interaction and identified E-cadherin cleavage sites for HtrA. Mass-spectrometry-based proteomics and Edman degradation revealed three signature motifs containing the [VITA]-[VITA]-x-x-D-[DN] sequence pattern, which were preferentially cleaved by HtrA. Based on these sites, we developed a substrate-derived peptide inhibitor that selectively bound and inhibited HtrA, thereby blocking transmigration of *H. pylori.* The discovery of HtrA-targeted signature sites might further explain why we detected a stable 90 kDa NTF fragment during *H. pylori* infection, but also additional E-cadherin fragments ranging from 105 kDa to 48 kDa in *in vitro* cleavage experiments. In conclusion, HtrA targets E-cadherin signature sites that are accessible in *in vitro* reactions, but might be partially masked on epithelial cells through functional homophilic E-cadherin interactions.

Chronic infections of humans by the pathogen and class-I carcinogen *Helicobacter pylori* (*H. pylori*) have been associated with several gastric disorders ranging from chronic gastritis and ulceration to lymphoma of the mucosa-associated lymphoid tissue (MALT) system and gastric cancer^1^,^2^. As the first target tissue, *H. pylori* persistently colonises the epithelial lining of the human stomach, which represents an important barrier against toxins and pathogens. The organisation and maintenance of the gastric epithelium as a protective barrier require functional adherens junctions with the classical cadherin Cdh1 (E-cadherin) as a key molecule. Structurally, E-cadherin consists of five extracellular repeats (EC1-EC5), a single transmembrane domain (TMD) and an intracellular domain (IC). E-cadherin mediates intercellular adhesions between epithelial cells through dynamic, calcium-dependent, homophilic *cis-* and *trans-*interactions of its ectodomains^3^. The IC domain of E-cadherin binds to cellular proteins such as β-catenin, γ-catenin and p120-catenin, and bridges E-cadherin via α-catenin to the intracellular actin cytoskeleton. Beside their function in the adhesive E-cadherin complex, β-catenin and p120-catenin are also implicated in nuclear cancer-associated target gene expression. When released from the intracellular E-cadherin domain, β-catenin can accumulate in the cytoplasm and interfere with Wnt signalling, but can also translocate into the nucleus, where it can form a complex with transcription factors of the T-cell factor/lymphoid enhancer factor-1 (Tcf/LEF-1) family^4^,^5^. A similar role has been described for p120-catenin, which can relieve Kaiso-dependent transcriptional repression in the nucleus^6^,^7^. Therefore, an intact E-cadherin complex plays a significant role in cell-to-cell adhesion and prevention of invasive growth and metastasis of many tumour types^8^.

*H. pylori*-associated metastatic gastric cancer (GC) is the leading cause of cancer-related deaths worldwide because effective treatment strategies are missing. Both the diffuse and intestinal GC types have been correlated with an altered functionality of the E-cadherin complex. The most prominent mechanisms in the gastric system include (i) loss-of-function mutation of the *cdh1* gene^9^,^10^, (ii) epigenetic down-regulation of E-cadherin expression^11^,^12^ and/or (iii) shedding of the E-cadherin ectodomain^13^. The proteases involved and their substrate cleavage patterns are still not entirely known. E-cadherin ectodomain cleavage was originally observed in breast cancers^14^. The soluble ~90 kDa N-terminal E-cadherin fragment (NTF) has been associated with a broad range of cancers and correlates with the grade, number and recurrence of certain tumours^15^. E-cadherin-cleaving enzymes comprise various host cell metalloproteases (e.g. MMP-3, -7, -9, ADAM-10, -15)^16^,^17^,^18^,^19^ and the protease HtrA (high temperature requirement A), both of bacterial and human origin^20^,^21^.

Serum levels of soluble E-cadherin are increased in the intestinal GC-type and might serve as a biological marker^22^. It has been previously shown that *H. pylori* induces strong E-cadherin shedding, which leads to the disruption of adherens junctions^23^. *H. pylori*-activated ADAM-10 (a disintegrin and metalloprotease) has been suggested to be responsible for the cleaving of E-cadherin on host cells^24^. However, we found that inhibition of a wide range of matrix metalloproteases (MMPs) together with siRNA-mediated downregulation of ADAM-10 only slightly decreased *H. pylori*-mediated E-cadherin shedding, while a major contribution of *H. pylori* HtrA was detected^21^. In fact, HtrA is a secreted bacterial serine protease that, together with host proteases, directly targets E-cadherin exposed on the surface of gastric epithelial cells^21^. The biological significance of HtrA-mediated E-cadherin cleavage has also been shown for the Gram-negative pathogens *Campylobacter jejuni* (*C. jejuni*), enteropathogenic *Escherichia coli* (EPEC) and *Shigella flexn*eri of the gastrointestinal tract^20^,^25^,^26^, indicating that E-cadherin represents a primary target in bacterial pathogenesis.

HtrA has already been intensively investigated in *E. coli* expressing the three HtrA homologues DegP, DegQ and DegS. The main function of bacterial HtrA is protein quality control and degradation of misfolded proteins in the periplasm, enhancing bacterial survival under stress conditions. In fact, *H. pylori* HtrA is highly active under extreme conditions^27^, indicating that it is important for bacterial physiology in the environment of the stomach. This is supported by the finding that the *htrA* gene is essential and cannot be deleted or mutagenised in the *H. pylori* genome^21^,^28^,^29^. The observation that secreted *H. pylori* HtrA directly affects the integrity of the gastric epithelium via E-cadherin ectodomain shedding implies that truncated E-cadherin could also interfere with *H. pylori*-induced signalling in GC.

Previous studies investigating MMP-mediated E-cadherin cleavage and Edman sequencing of E-cadherin fragments have suggested the residue pattern L^585↓^S^↓^D^587^ (CDH1, P12830) in the EC4 domain as a target site^17^. In another study, P^700↓^V^701^ was proposed as an MMP cleavage site^19^. Whether *H. pylori* HtrA targets the same sites or prefers another amino acid sequence is unknown, but this information is crucially important for understanding the HtrA-E-cadherin interaction and developing HtrA inhibitors.

In the present study, we investigated the molecular mechanism of the HtrA-E-cadherin interaction. We discovered that E-cadherin signature motifs function as target sites for the proteolytic degradation by HtrA, leading to a defined fragmentation pattern of E-cadherin. As HtrA-mediated E-cadherin shedding may possess cancer-promoting properties, we used the identified signature sites to design the first substrate-based peptide inhibitor to impede HtrA-mediated E-cadherin cleavage and *H. pylori* transmigration across a polarised monolayer.

## Results

### *H. pylori* HtrA mediates different cleavage patterns of E-cadherin *in vitro* and on gastric epithelial cells

**Figure 1:**
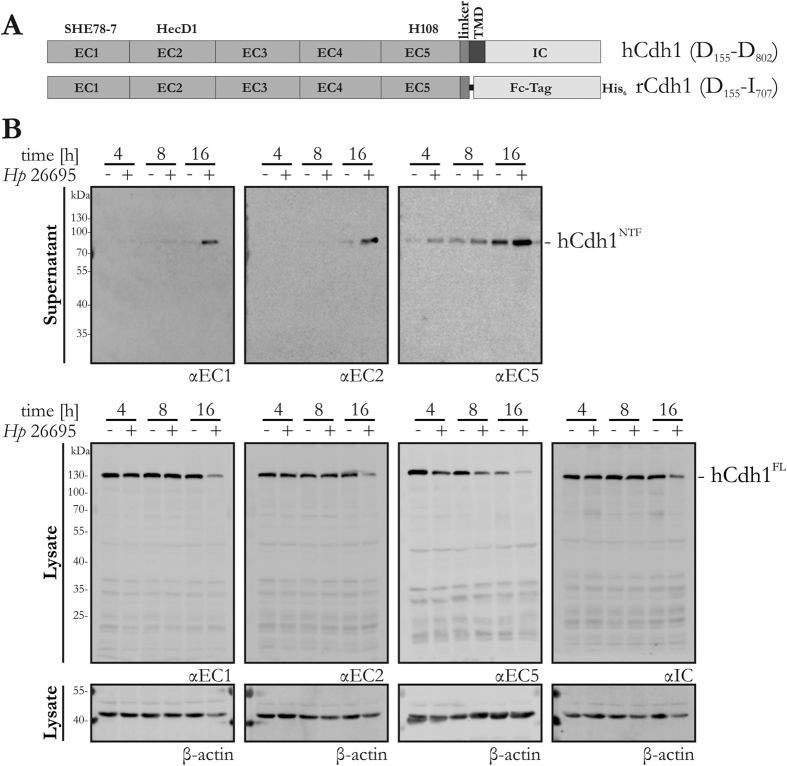
Formation of a stable 90 kDa E-cadherin fragment after infection with *H. pylori*. (**A**) Domain structures of human endogenous E-cadherin (hCdh1) and recombinant E-cadherin (rCdh1). E-cadherin consists of five extracellular domains (EC1–EC5) and a linker region. hCdh1 contains a transmembrane domain (TMD) and an intracellular domain (IC). rCdh1 is expressed as a Fc-tag/His_6_ fusion protein. The monoclonal antibodies recognise the EC1 (SHE78-7) or the EC2 domain (HecD1). The polyclonal antibody H108 is directed against the EC5 domain. (**B**) NCI-N87 cells were infected with *H. pylori* at a MOI of 100 (+) for the indicated time periods or left untreated (−). E-cadherin fragments in the supernatant (upper panels) and in the lysates (lower panels) of infected cells were detected using antibodies recognising the EC1, EC2, EC5, or the IC domains. β-actin was detected as a control.

Several pathogenic bacteria secrete the HtrA protease, which cleaves off the extracellular NTF domain of E-cadherin on epithelial host cells to open intercellular junctions^20^,^21^,^25^,^26^. However, it is completely unknown how HtrA interacts with E-cadherin at the molecular level. The aim of this study was the identification of HtrA cleavage sites as the basis for the future generation of potential pharmacological tools. Mature human E-cadherin (hCdh1) is expressed as a 125 kDa glycoprotein, which consists of the five extracellular domains EC1–EC5, a linker region, a single transmembrane domain (TMD) and an intracellular (IC) domain (Fig. 1A, hCdh1 D^155^–D^882^). Several antibodies are available that selectively recognise individual domains of the E-cadherin protein. SHE78-7 detects the EC1 domain, the HecD1 antibody recognises the EC2 domain and H108 binds to the EC5 domain^19^,^30^. The specificity and selectivity of these antibodies was confirmed by using deletion mutants of recombinant E-cadherin lacking the EC1, EC1–2, EC1–3, EC4–5 and EC5 domains (see Supplementary Fig. S1A, Fig. S1B, Table S1). To investigate the fragmentation of E-cadherin, NCI-N87 cells were infected with *H. pylori* for the indicated time periods and aliquots of the supernatants were analysed for E-cadherin fragments containing the EC1, EC2 or EC5 domain. After 16 h of infection, the anti-EC1 antibody detected a strong increase in the 90 kDa NTF fragment of E-cadherin (hCdh1^NTF^). We observed a similar picture for the anti-EC2 antibody. When the supernatant was analysed with the anti-EC5 antibody, the hCdh1^NTF^ fragment enriched during *H. pylori* exposure was already visible after 4 h of *H. pylori* exposure and the levels increased during the 16 h of infection (Fig. 1B, upper panels). Further fragments were not detectable, even after longer exposures of the membranes (not shown). These results indicate that *H. pylori* induces production of a stable 90 kDa hCdh1^NTF^ fragment which contains the EC1 to EC5 domains. Although *H. pylori* adhered slightly more strongly to AGS than to MKN-28 or NCI-N87 cells (see Supplementary Fig. S1C), the formation of a stable 90 kDa fragment of E-cadherin in the supernatants was reproducibly detected for all cell lines (Fig. 1B, see Supplementary Fig. S1D). Corresponding to the increase in the 90 kDa hCdh1^NTF^, we observed a significant drop in signals for the 125 kDa full-length E-cadherin protein (hCdh1^FL^) in lysates of *H. pylori*-infected NCI-N87 cells (Fig. 1B, middle panels). A slight decrease in hCdh1^FL^ could be detected using the anti-EC1, anti-EC2 and anti-EC5 antibodies during early time periods of *H. pylori* infections and an obvious loss of hCdh1^FL^ was visible after 16 h. An anti-IC antibody confirmed these results, with β-actin as loading control (Fig. 1B, lower panels).

**Figure 2:**
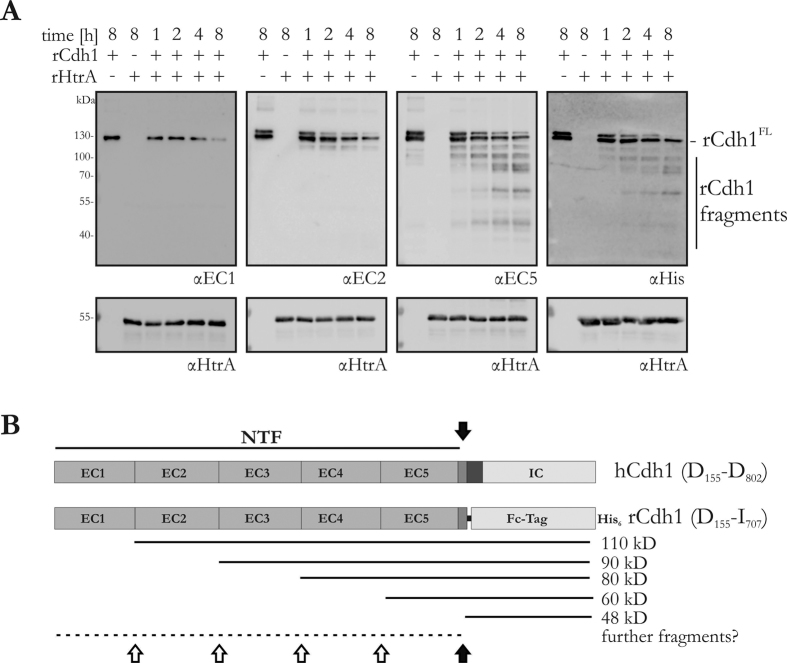
HtrA-mediated fragmentation of E-cadherin in *in vitro* cleavage experiments. (**A**) 100 ng recombinant E-cadherin was incubated with 200 ng recombinant HtrA at 37 °C for the indicated time periods. E-cadherin fragments were detected using antibodies recognising the EC1, EC2 or EC5 domains, or the C-terminal His_6_ tag. HtrA is shown as a control. (**B**) Detected E-cadherin fragments after infection with *H. pylori* (hCdh1) or *in vitro* cleavage (rCdh1). Putative cleavage sites are indicated by an open arrow. The black arrow indicates the possible cleavage site leading to EC5-containing fragments.

E-cadherin forms functional *cis*- and *trans*-interactions between the extracellular domains of two neighbouring cells^3^ which could overlay additional cleavage sites. Therefore, we examined fragment formation using a recombinant E-cadherin chimera (rCdh1 D^155^–I^707^). rCdh1 contains the five EC domains and the linker region (A^698^–I^707^), but lacks the TMD. Instead of the IC domain, rCdh1 is fused to the Fc region and a His_6_ tag (Fig. 1A, rCdh1 D^155^–I^707^). Incubation of rCdh1 with recombinant *H. pylori* HtrA (rHtrA) for the indicated time periods showed a different cleavage pattern compared to the *H. pylori* infection experiments. Using the anti-EC1 antibody, we only detected the loss of the full-length E-cadherin protein (rCdh1^FL^) (Fig. 2A). Even after long exposure, the Western blots did not reveal further fragments, suggesting an immediate loss of the EC1 domain (see Supplementary Fig. S2). The anti-EC2 antibody detected an additional high molecular weight (~110 kDa) fragment (Fig. 2A, see Supplementary Fig. S2). A ladder of putative E-cadherin fragments of approximately 105 kDa, 90 kDa, 60 kDa and 48 kDa was observed using the anti-EC5 antibody (Fig. 2A). Similar fragments appeared when an anti-His tag antibody was applied (Fig. 2A, see Supplementary Fig. S2), indicating that fragmentation of E-cadherin begins at the N-terminus with the cleaving of individual EC domains (Fig. 2B). According to the fragment sizes observed during *H. pylori* infections and in *in vitro* cleavage experiments, we suggest that E-cadherin is N-terminally and C-terminally processed by HtrA. Upon *H. pylori* infection, a stable 90 kDa NTF originating from hCdh1 is formed, while in *in vitro* cleavage experiments, rCdh1 is mainly N-terminally processed resulting in a series of different fragments (Fig. 2B).

### Identification of HtrA cleavage sites in E-cadherin

**Figure 3:**
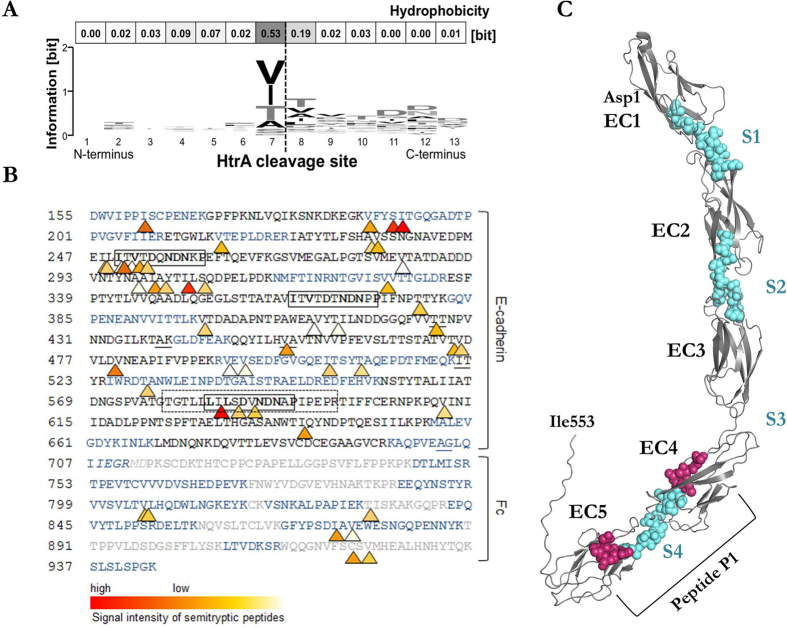
Identification of signature sites. (**A**) Sequence logo and hydrophobicity of HtrA cleavage sites in E-cadherin. The height of the amino acid one-letter codes illustrates their relative observed frequency. The local cleavage site residue pattern is [VITA]↓[VITA]-x-x-D-[DN]. (**B**) HtrA-cleavage sites of E-cadherin peptides. The triangles denote the non-tryptic cleavage positions found by label-free mass spectrometry-based proteomic analysis of combined HtrA/tryptic digests. Their colour illustrates the signal intensity of the detected peptides. The amino acids drawn in blue designate fully tryptic peptides. Cleavage sites found by N-terminal Edman sequencing are shown as underlined residues. The amino acids drawn in grey are not a part of E-cadherin (linker, Fc domain of IgG1). The cadherin domain signature [LIV]-x-[LIV]-x-D-x-N-D-[NH]-x-P is highlighted within a box. The P1 peptide is shown in a dashed box. (**C**) Comparative model of human E-cadherin (template PDB-ID: 3Q2V chain A) with its five EC domains. The experimentally detected signature sequence stretches are shown as coloured balls (turquoise) to contrast them to the grey ribbon representing the EC domains. Different colouring of the flanking regions (in magenta) of S4 shows the overhanging parts of peptide P1.

To identify the HtrA cleavage sites in E-cadherin, we performed a label-free mass spectrometry-based proteomic analysis of HtrA/trypsin digests of rCdh1. Overall, we detected 128 peptides of E-cadherin origin, among which we identified 46 semi-tryptic peptides that were processed solely by HtrA at the non-tryptic cleavage site. These peptides were commonly cleaved after the residues I (37%), V (28%), T (17%), A (9%) and S (9%). Sequence analysis revealed that HtrA cleavage takes place favourably between hydrophobic amino acids (Fig. 3A,B). We also performed N-terminal Edman sequencing of rCdh1 cleavage products to validate the cleavage pattern obtained from the same recombinant Cdh1 protein. These results agree with the mass spectrometric analysis. Four of five detected N-terminal cleavage sites (T^↓^T, A^↓^K, V^↓^A, I^↓^T, A^↓^G) match the proteomic cleavage site pattern [VITA]^↓^[VITA]-x-x-D-[DN], which are located within the four calcium-binding sites and which we have named “signature sites” S1, S2, S3 and S4 (Fig. 3C). The signature site S3 between EC3 and EC4 was less frequently targeted by HtrA. This consensus cleavage pattern for HtrA-catalysed proteolysis is related to that of its *E. coli* orthologue DegP, which also favourably cleaves between hydrophobic residues^31^. We further observed that HtrA cleaved most frequently in the fourth signature sequence of E-cadherin (Fig. 3B). Its I^↓^L cleavage site was detected with the highest intensity of all peptides.

### Identification of substrate-derived inhibitory peptides

**Figure 4:**
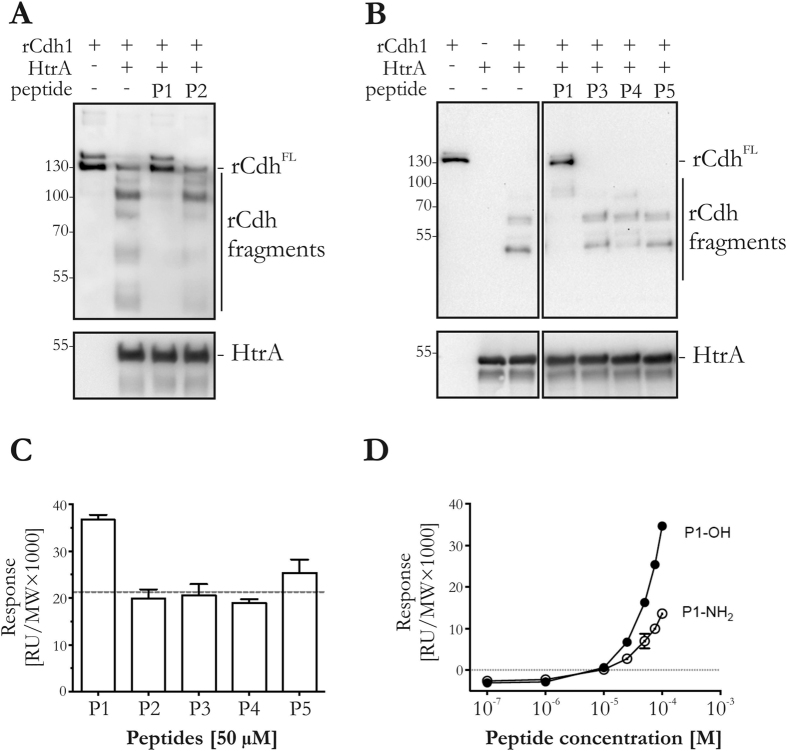
Development of a signature site-based peptide inhibitor. (**A**,**B**) E-cadherin cleavage by HtrA in the presence of 100 μM peptides P1, P2, P3, P4 and P5. P1 efficiently inhibited HtrA-mediated E-cadherin cleavage. Different sections of the same membrane with samples analysed under the same experimental conditions are shown in (**B**). The original Western blot image is shown in Fig. S6. (**C**) Measurement of direct peptide binding to HtrA by SPR. The dotted line represents the activity threshold. (**D**) The P1 peptide with a free carboxyl C-terminus (P1-OH, filled circles) evoked a greater SPR response than its amide counterpart (P1-NH_2_, open circles).

Since deletion or mutation of the *htrA* gene in the *H. pylori* genome is not possible, we aimed at the generation of a peptide-based E-cadherin cleavage inhibitor. We synthesised five peptides (P1−P5) around the cleavage signature sequence between E-cadherin domains EC4 and EC5 (positions L^583^–P^593^) (Fig. 3C, see Supplementary Fig. S4A). The 21-residue peptide P1 (see Supplementary Fig. S3A, Fig. S4A) effectively inhibited *in vitro* HtrA-mediated E-cadherin cleavage (Fig. 4A). Shorter peptides (see Supplementary Fig. S4A) did not exhibit any suppressive effect (Fig. 4A,B). The direct binding affinities of the peptides to immobilised HtrA were measured by surface plasmon resonance (SPR). Peptide P1 showed strong binding, while only one other cleavage-sequence peptide (P5) evoked a positive binding response above the significance threshold (Fig. 4C). N-terminal truncation of the P1 peptide led to a loss of enzyme inhibition, indicating that the N-terminal part of P1 is important for substrate recognition by HtrA. In a competition experiment, we analysed the short peptide P1_NT, which contains the hydrophobic amino acid stretch TGTLLLI. As controls, P1_NT (TG) and P1_NT (GT) were included as scrambled 7-residue peptides (see Supplementary Fig. S4A). None of these peptides blocked HtrA-induced E-cadherin cleavage or interfered with P1-mediated HtrA inhibition (see Supplementary Fig. S4B). We then substituted key amino acids in the C-terminal (P1_CT_mut_) or N-terminal (P1_NT_mut_) regions with glycine (see Supplementary Fig. S4A). Both P1_CT_mut_ and P1_NT_mut_ only partially inhibited HtrA-mediated E-cadherin cleavage (see Supplementary Fig. S4C). Therefore, we postulate that the 21-residue P1 peptide is required for efficient HtrA inhibition. We also synthesised P1 with a C-terminal alpha-amide and subjected both P1 variants (P1-OH, P1-NH_2_) to SPR analysis. Although both peptides exhibited concentration-dependent binding (see Supplementary Fig. S3B, Fig. S3C), the P1-peptide with the natural C-terminal carboxylic acid evoked stronger SPR signals, reflecting its binding capacity to HtrA (Fig. 4D). This result is in agreement with a study by Krojer *et al*., who compared peptide substrates of the *E. coli* orthologue DegP^32^.

**Figure 5:**
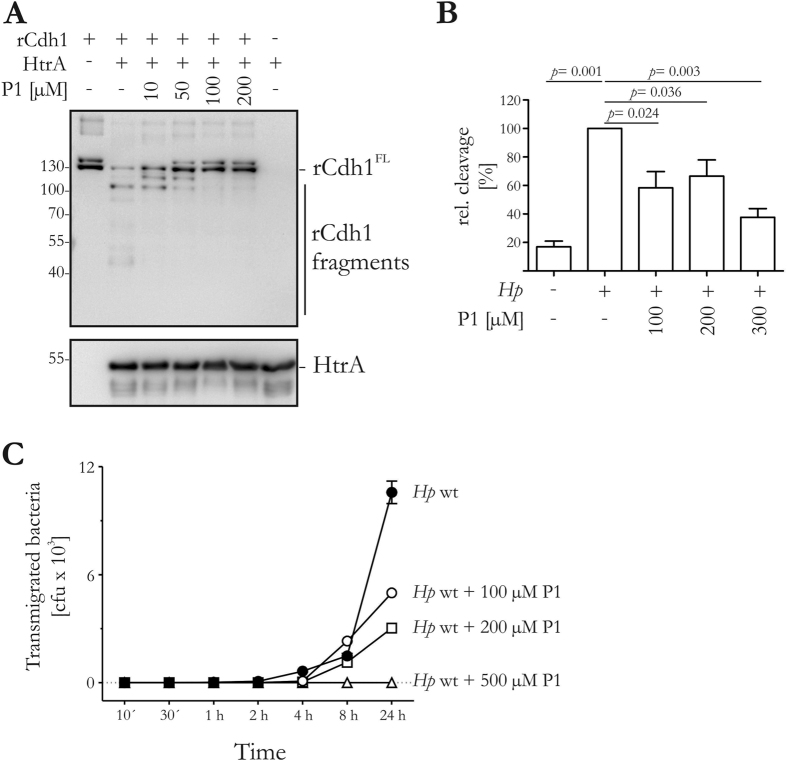
Inhibition of HtrA-mediated E-cadherin cleavage. (**A**) 50 ng rCdh1 was incubated with 200 ng HtrA and indicated concentrations of P1. rCdh1 fragments and HtrA were detected using specific antibodies. (**B**) Transfected AGS cells expressing hCdh1 were infected with *H. pylori* (*Hp*) or left untreated (–) for 16 h. 50 μl aliquots of the supernatants were separated by SDS PAGE and blotted onto membranes. The 90 kDa hCdh1^NTF^ fragment was detected by Western blots. The relative amount of hCdh1^NTF^ fragment was quantified by blot densitometry from three independent experiments. These results are given as arbitrary units with the hCdh1^NTF^ fragment levels produced in response to *H. pylori* which were not treated with P1 set to 100%. (**C**) Polarised MKN-28 cells grown on transwell filters were infected with *H. pylori* in the presence and absence of peptide P1 at different concentrations (100 μM, 200 μM, 500 μM). Bacteria that had transmigrated across the MKN-28 monolayer on the filter were counted (*n* = 3, *mean* ± *std dev*).

Peptide P1 strongly inhibited HtrA-mediated rCdh1 cleavage in a concentration-dependent manner (Fig. 5A). To investigate the bioactivity of P1, we quantitated the inhibition of HtrA in *H. pylori*-infected AGS cells that were transfected with hCdh1^FL^. We observed a significant dose-dependent decrease in the formation of the hCdh1^NTF^ (Fig. 5B) in comparison with the corresponding loading controls (see Supplementary Fig. S4D). This effect was also observed in *H. pylori* infections of several other cell lines and at different infection doses (data not shown). We then analysed the epithelial transmigration of *H. pylori* in a transwell filter assay. Partially polarised MKN-28 cell monolayers were infected and the transmigration of E-cadherin-cleaving *H. pylori* was determined over time. Polarisation of MKN-28 cells requires functional tight junctions, which can be monitored by the measurement of the transepithelial electrical resistance (TEER). Corresponding to previous studies^21^,^25^,^33^,^34^, MKN-28 cells showed partial polarisation, as reflected by a resistance of ∼150 Ω/cm^2^ after 14 days of cultivation (see Supplementary Fig. S5A). Infections with *H. pylori* (see Supplementary Fig. S5B) did not significantly alter the polarisation of host cells. However, we observed a strong concentration-dependent reduction of *H. pylori* transmigration by P1 (Fig. 5C), indicating that P1 efficiently blocks HtrA-mediated E-cadherin cleavage. These results confirm the bioactivity of P1 as the first inhibitory peptide blocking bacterial HtrAs.

## Discussion

E-cadherin is an important cell adhesion molecule and tumour suppressor. Ectodomain shedding and an increase in soluble E-cadherin is associated with several cancer types, such as breast cancer, pancreatic cancer and GC^15^. In recent years, several E-cadherin-cleaving proteases have been described. MMPs, such as MMP-7 (matrilysin)^35^ and MMP-3 (stromelysin-1)^36^, or proteases such as ADAM-10^37^ are examples that cleave off the extracellular E-cadherin domain. In *H. pylori*-infected cells, activated ADAM-10 (a disintegrin and metalloprotease) has been suggested as a protease responsible for the cleavage of E-cadherin on host cells^24^. Furthermore, *H. pylori* induces the expression of several MMPs, including MMP-3 and MMP-7, which can also contribute to E-cadherin shedding^21^,^24^. Recently, HtrA was added to this collection as the first E-cadherin-cleaving protease expressed by *H. pylori*^21^. Generally, knowledge of *H. pylori* proteases is still rare and no additional *H. pylori* protease has yet been identified as an E-cadherin-cleaving protease. *H. pylori* expresses an active collagenase (Hp0169), which is crucially important for *in vivo* colonisation^38^, but this has not been tested for E-cadherin cleavage. Previous studies have predicted several putative *H. pylori* proteases with an extracellular localisation^39^,^40^. Among these, Hp0506 is required for bacterial cell shape and pole formation^41^, but, like Hp0657 and Hp1012, it does not target E-cadherin as a substrate^21^. Since E-cadherin acts as an important suppressor of carcinogenesis, increased understanding of the direct interaction between HtrA and E-cadherin is required. Therefore, we report here on a comprehensive cleavage site map for E-cadherin and the development of an effective peptide inhibitor based on an HtrA-targeted substrate sequence.

**Figure 6:**
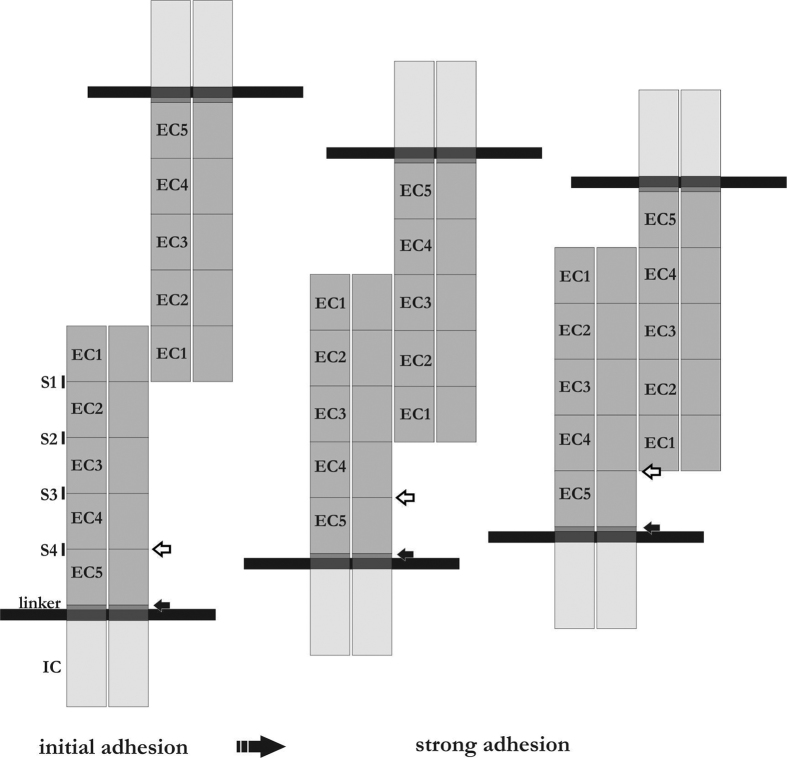
Model of the HtrA-induced E-cadherin cleavage pattern. E-cadherin contains four signature motifs, of which HtrA preferentially cleaves the three sequences S1, S2 and S4 ⇐ *in vitro*. An additional cleavage site might be located in the linker region (black arrow). On epithelial cells, the E-cadherin ectodomains interact in *cis* and in *trans* forming functional cell-to-cell adhesions. The extent of overlapping domains determines the accessibility of cleavage site for HtrA.

For MMPs, the amino acid sequence L^585↓^S^↓^D^587^ in the EC4 domain and P^700↓^V^701^ have been suggested as putative cleavage sites^17^,^19^. In our study, we identified the [LIV]-x-[LIV]-x-D-x-N-D-[NH]-x-P sequence pattern in the E-cadherin molecule as a target for *H. pylori* HtrA, which is in line with the L^585↓^S^↓^D^587^ cleavage site for MMPs^17^. This sequence pattern is located in three out of four calcium-binding regions of the E-cadherin molecule, which we have designated as signature sites S1, S2, S3 and S4. The accessibility of E-cadherin signature sites as cleavage sites for *H. pylori* HtrA and possibly for *H. pylori*-regulated MMPs might explain why we observed different fragmentation patterns in *H. pylori* infections and *in vitro* (Fig. 6). We included several different gastric epithelial cell lines in our study, which might be differentially colonised by *H. pylori*. It had been previously shown that *H. pylori* infection of MKN-28 cells induces the formation of the stable 90 kDa NTF^20^,^21^. Although AGS cells showed a slightly stronger binding of *H. pylori* in comparison to MKN-28 and NCI-N87 cells, an unaltered fragmentation pattern was detected, as reflected by the stable 90 kDa NTF. These data indicate that possible differences in *H. pylori* adherence to host cells do not influence E-cadherin shedding by secreted HtrA, while *cis* and/or *trans* interactions of E-cadherin might be important. The interaction of the ectodomains at the molecular level is still not fully understood, but has been described as a highly dynamic process^3^. It has been suggested that the EC1 domain forms an initial weak intercellular adhesion, while strong adhesion requires at least EC1–3^42^ (Fig. 6). In *in vitro* cleavage experiments, rCdh1 likely exists as a monomer that presents all signature sites as possible cleavage sites (Fig. 6). In contrast, epithelial cells form functional E-cadherin-mediated adhesions through *cis* and *trans* interactions of the extracellular E-cadherin domains. This tight binding might overlay the signature sites S1 to S3 and probably S4^42^,^43^, or induce changes in their structural accessibilities. In this model, *H. pylori* could still target the S4 signature site or an undiscovered cleavage site in the linker region leading to the formation of the 90 kDa fragment (Fig. 6). Due to the glycosylation of E-cadherin, the size of the E-cadherin sequence from D^155^ to the S4 site is not definitively assessable. Therefore, we cannot exclude the possibility that HtrA also targets a sequence in the linker region near the transmembrane domain which was not detectable after tryptic digests. In conclusion, we have identified signature sites in the E-cadherin protein that are cleaved by *H. pylori* HtrA leading to the fragmentation of E-cadherin, depending on the *cis* and *trans* interactions between the extracellular domains.

Since an *htrA* knock-out *H. pylori* mutant is still lacking, we focused on pharmacological inhibition of *H. pylori* HtrA. Several small molecule compounds targeting *H. pylori* HtrA have been successfully developed which can function as lead structures^21^,^44^,^45^,^46^. Here, we present the first substrate-derived peptide that efficiently binds and inhibits *H. pylori* HtrA. The P1 peptide TGTLLLILSDVNDNAPIPEPR corresponds to the E-cadherin sequence T^578^–R^598^ surrounding the signature site S4. The P1 peptide binds with high specificity and efficiently blocked E-cadherin cleavage *in vitro*. The inhibitory effect of P1 in *H. pylori* infections was weaker, but still significant at higher concentrations. The bioactivity of P1 was further confirmed by the inhibition of *H. pylori* transmigration. Although not investigated in this study, the P1 peptide might be captured by other E-cadherin binders or might be degraded. Although our data provide direct evidence for HtrA inhibition by the P1 peptide, which corroborates the idea that HtrA enzymatic activity is critical for E-cadherin cleavage, an optimisation of substrate-based inhibitors is still necessary.

E-cadherin could play a central role in *H. pylori*-dependent invasiveness of tumour cells, which is indicated by the finding that the *H. pylori* virulence factor cytotoxin-associated gene A (CagA) can interact with the IC of E-cadherin, interfering with its tumour-suppressing function^47^,^48^. It would be compelling to investigate if truncated E-cadherin still binds the intracellular catenin complex or CagA, and if catenin signalling is affected. Importantly, the soluble 90 kDa NTF fragment of E-cadherin does not only serve as a biomarker for GC^49^, but can also impair cell adhesions in a paracrine manner^35^. These data imply that gastric E-cadherin ectodomain shedding is also important in *H. pylori*-dependent carcinogenesis. In conclusion, our results provide an inspiring novel entry point for innovative anticancer drug discovery.

## Methods

### Cell culture and infection experiments

The human gastric adenocarcinoma cell line AGS is E-cadherin-negative and was derived from an adenocarcinoma of the stomach of a 54 year-old Caucasian female (ECACC, 89090402). AGS cells expressing hCdh1 were generated by transfection using a plasmid containing E-cadherin wild-type cDNA^50^ which has been kindly provided by Rohan D. Teasdale (University of Queensland, Australia). E-cadherin-positive MKN-28 cells (JCRB, 0253, obtained from the Max-Planck Institute for Infection Biology) were originally described as being isolated from a moderately differentiated tubular adenocarcinoma and have been frequently used to study E-cadherin-dependent processes^20^,^21^,^23^,^25^. The gastric epithelial NCI-N87 cells (ATCC, CRL-5822) were included as an additional E-cadherin-expressing cell line^24^ and were derived from metastasis in the liver. All cells have been described previously^24^,^51^ and were grown in RPMI 1640 medium containing 4 mM glutamine (Invitrogen) and 10% FCS (Sigma) in a humidified atmosphere at 37 °C. *H. pylori* wild-type strains P12^52^ and *Hp*26695^53^ were cultured on agar plates containing 10% horse serum under microaerophilic conditions for 48 h at 37 °C before infection experiments. Cells were infected with *H. pylori* at a MOI of 100. Adherence of *H. pylori* was quantitated in a cfu (colony-forming unit) assay. Briefly, bacteria were suspended in culture medium, added to the cells at a MOI of 100 and co-incubated with host cells for 16 h. The number of cfu was determined by growth on agar plates for 72 h. To investigate the influence of the P1 inhibitor peptide on bacterial transmigration, transwell infections were performed in the presence or absence of 100, 200 or 500 μM peptide. The human gastric adenocarcinoma cell line MKN-28 was grown to confluence on transwell filters and polarised for 14 days. Establishment of functional tight epithelial cell monolayers was further confirmed by measuring the transepithelial electrical resistance (TEER). Infection of these cells was carried out with bacteria at a MOI of 50 for the indicated periods of time per experiment. The number of transmigrated bacteria was determined as cfu by taking aliquots from the basal chambers^21^,^54^. All infection assays were done in triplicate.

### Western blotting

Proteins were separated by SDS PAGE and analysed by Western blot using specific antibodies against HtrA^21^, the extracellular domains EC5 (H108, Santa Cruz Biotechnology and ab40772, Abcam), EC1 (SHE78-7, Invitrogen) and EC2 (HecD1, Calbiochem) of E-cadherin, or the intracellular domain (24E10, Cell Signaling). His_6_-tagged proteins were analysed using an anti-His_5_-antibody (Qiagen). GAPDH (Cell Signaling) and β-actin (Sigma) were detected as loading controls. Where indicated, signals of protein bands were quantified using the ImageLab software (BioRad). Graphical presentation and statistical evaluations (Student’s *t*-test) were performed with GraphPad Prism 5 and *p*-values of ***p ≤ 0.001, **p ≤ 0.01 and *p ≤ 0.05 were considered statistically significant.

### *In vitro* HtrA proteolytic activity

Purification of recombinant *H. pylori* HtrA and *in vitro* cleavage assays have been described elsewhere^21^,^39^. Briefly, 200 ng recombinant HtrA was incubated with 50 ng recombinant hCdh1 (R&D Systems) in 50 mM HEPES pH 7.4 and 100 μM EDTA for 16 h (or for the indicated time periods) at 37 °C. Where indicated, test peptides were added.

### Samples for combined HtrA and tryptic digestion

Tryptic digestion was performed on three different samples (sample volume: 200 μl). Sample 1 contained human recombinant E-cadherin Fc His_6_ (22.5 ng/μl E-cadherin D^155^–I^707^, R&D Systems) in 50 mM HEPES buffer (HEPES buffer 1 M solution, pH 7.3, Fisher Scientific), 0.5 mM MgCl_2_ (Sigma-Aldrich Chemie GmbH) and 1 mM CaCl_2_ (Sigma-Aldrich Chemie GmbH, pH 7.3). Sample 2 contained HtrA (2.5 ng/μl in 50 mM HEPES buffer, pH 7.3). Sample 3 included HtrA and human recombinant E-cadherin Fc His_6_ at a ratio of 1:10 (2.5:22.5 ng/μl in 50 mM HEPES buffer, pH 7.3). All samples were incubated for 12 h at 37 °C in a horizontal shaker. Samples 1 and 2 were applied in sextuplicate, and sample 3 was done in triplicate. Finally, 2 μl of sequencing-grade modified porcine trypsin was added (stock solution of 50 ng/μl in 50 mM HEPES, 1 mM CaCl_2_, pH 7.3, Promega). Protease digestion was carried out overnight at 37 °C in a horizontal shaker. After tryptic digestion, Samples 2 and 1 were mixed at a ratio of 1:10 to obtain Sample 4. Peptides were desalted, purified and concentrated using C18 microcolumns OMIX tips (Agilent Technologies). After lyophilisation, peptides were stored at –20 °C.

### Nanocapillary HPLC with automated on-line fraction spotting onto MALDI target plates

Peptides were separated by reverse-phase high-performance liquid chromatography using an EASY-nLC system (Proxeon, now Thermo Fisher Scientific) with mobile phase A: 0.1% trifluoroacetic acid (TFA) in water; mobile phase B: 0.1% TFA in acetonitrile; flow rate of 300 nl/min. Lyophilised peptides were dissolved in 23 μl of buffer A, of which 18 μl were loaded onto the column (inner diameter = 75 μm; length = 15 cm; filled with ReproSil-Pur C18 AQ, 3 μm, 120 Å beads; Dr. Maisch GmbH). The peptides were eluted with a gradient of 5–33% B for 62 min, 33–48% B for 15 min, 48–100% B for 2 min and 100% B for 10 min. The column was then equilibrated with 5% B for 20 min before the next sample was analysed. Eluting fractions were mixed with a solution of 3 mg/ml α-cyano-4-hydroxycinnamic acid, 187.5 pmol/ml of each of the four internal standard peptides ([des-Arg^9^]-bradykinin, neurotensin, angiotensin I and adrenocorticotropic hormone fragment 1–17; all from Sigma), 0.1% TFA and 70% acetonitrile in water and deposed on a blank MALDI target plate (416 spots per sample) using an online SunCollect system (SunChrom). The final concentration of each internal peptide standard was 50 fmol per spot.

### Mass spectrometric analysis

MALDI-TOF/TOF analysis was carried out with a 4800 MALDI TOF/TOF Analyzer (AB SCIEX). All spectra were acquired with a solid-state laser (355 nm) at a laser repetition rate of 200 Hz. After measuring all samples in the MS mode, a maximum of 12 precursors per spot were automatically selected for subsequent fragmentation by CID using the mass spectrometer control software (4000 Series Explorer V3.7, AB SCIEX). The resulting spectra were processed and analysed using the Global Protein Server Workstation version 3.6 (GPS Explorer, AB SCIEX), which uses internal MASCOT version 2.1 (Matrix Sciences) software for matching MS and MS/MS data against databases of *in silico*-digested proteins. Both fully tryptic and semi-tryptic peptides were taken into account. MS/MS data was searched against a database consisting of *H. pylori* HtrA (UniProt ID: G2J5T2)^55^, glutathione S-transferase class-mu 26 kDa isozyme from *Schistosoma japonicum* (UniProt ID: P08515), human recombinant E-cadherin Fc His_6_ and typical contaminants from recombinant protein expression (yielding a total of 27455 proteins). Possible contaminants included amino acid sequences of human keratins, *Escherichia coli* and proteins from foetal calf serum downloaded from UniProt (www.uniprot.org). The following analysis settings were used for the identification of peptides and proteins: (i) precursor tolerance: <15 ppm, (ii) MS/MS fragment tolerance: 0.5 Da, (iii) maximal missed cleavages: 1, (iv) one variable modification (oxidation of methionine). Peptides were considered correct calls if the confidence interval exceeded 95%. After MS acquisition, the data related to the individual peaks (fractions, intensities, mass-to-charge ratios) were loaded into the DeepQuanTR software^56^, which performed a normalisation of individual signal intensities to the internal standard peptides and an annotation (peptide identification and association with a parent protein). Normalised intensities for the individual peptides from all samples of each group (Samples 1–4) were used for the computation of DeepQuanTR peptide and protein scores, indicating the relative abundance of individual peptides and proteins in the sample groups.

### N-terminal sequencing

Edman sequencing was performed on an acid-etched glass fibre disk or on a PVDF membrane of an ABI Procise 494 sequencer. Prominent E-cadherin cleavage product bands of the HtrA cleavage assay were eluted and N-terminal sequencing was performed by Alphalyse A/S (Odense).

### Peptide synthesis and analytics

Peptide synthesis was performed on robotic solid-phase peptide synthesisers (Overture^TM^ and Symphony^TM^, Protein Technologies) utilising Fmoc-protected amino acids (AAPPTec) and Fmoc-Wang-resin (AAPPTec). Deprotection was performed for >2 min with 20% piperidine (Sigma-Aldrich Chemie GmbH) or 20% pyrrolidine (Sigma-Aldrich Chemie GmbH) in DMF (dimethylformamide, Sigma-Aldrich Chemie GmbH). A coupling reagent of amino acid, HCTU (O-(6-chloro-1-hydrocibenzotriazol-1-yl)-1,1,3,3-tetramethyluronium hexafluorophosphate, AAPPTec) and NMM (4-methylmorpholine, Fisher Scientific AG) in DMF was used for double coupling (min. 2 × 5 min), multiple washing steps after deprotection and double coupling. A cleavage reagent of TFA (2,2,2-trifluoroacetic acid, Fisher Scientific AG), H_2_O and TIPS (triisopropylsilane, Sigma-Aldrich Chemie GmbH) was used for automated cleavage. Peptides were isolated by using ice-cold diisopropylether (Sigma-Aldrich Chemie GmbH), rewashed there times, dried and stored at –20 °C. For peptide analytics, a linear gradient of 5–70% ACN/H_2_O(0.1% TFA) (acetonitrile, Sigma-Aldrich Chemie) over 25 min with a flow rate of 0.5 ml/min on a rpC18, 110 Å, 5 μm, 150 × 3 mm column (Macherey-Nagel) was used on a LC-20A HPLC instrument (Shimadzu). Mass identification was performed by a Shimadzu LCMS-2020 single-quad mass spectrometer (ESI+) at an interval of 300–1500 Da. Calculated molecular weights (*mw*, unit: Da), detected retention times (*R*_*t*_, unit: minutes) and observed masses (*m*+, unit: Da) were: TGTLLLILSDVNDNAPIPEPR (*mw* = 2248, *R*_*t*_ = 11.17, *m*+ = 541.80, 664.10, 750.35, 750.75, 1124.50, 1124.90, 1125.60), LLILSDVNDNAPIPEPR (*mw* = 1876, *R*_*t*_ = 9.93, *m*+ = 558.30, 643.90, 797.65, 881.60, 985.30, 965.75, 952.05, 1269.00, 1802.20, 1876.20), ILSDVNDNAPIPEPR (*mw* = 1650, *R*_*t*_ = 10.09, *m*+ = 1651.00), LSDVNDNAPIPEPR (*mw* = 1536, *R*_*t*_ = 9.25, *m*+ = 1537.00) and NDNAPIPEPR (*mw* = 1122, *R*_*t*_ = 8.79, *m*+ = 444.20, 498.45, 561.95, 562.75, 708.25, 893.40, 1008.45, 1122.50, 1123.50, 1124.40).

### Surface plasmon resonance (SPR)

Affinity studies were performed on a Sierra Sensors GmbH SPR-2 instrument. Measurements were performed at 25 °C with a flow rate of 25 μl/min. Carboxymethyl dextran matrix high-density sensor chips (SPR-2 Affinity Sensor HC, batch 10-BC-04-154-A, Sierra Sensors) were used to immobilise 20 μg/ml HtrA wild-type (wt) in 10 mM HEPES (HEPES buffer 1 M solution, pH 7.3, Fisher Scientific) by adding 198 μl amine coupling with an activation solution (200 mM N-ethyl-3-(3-dimethylaminopropyl)-carbodiimide [EDC] and 50 mM N-hydroxysuccinimide [NHS]). The SPR sensor surface was loaded with 100 μl protein and subsequently treated by injecting 192 μl of 1 M ethanolamine at pH 8.5 for inactivation of excess coupling groups. HBS-P (10 mM HEPES-buffered saline, 150 mM NaCl, 0.005% Tween) served as running and sample buffer for peptide measurements. Peptide samples were directly dissolved in HBS-P buffer. The experiments with the non-peptidic compounds were performed utilising HBS-PD running buffer (10 mM HEPES-buffered saline with 3% DMSO, 150 mM NaCl, 0.005% Tween).

### Comparative model of human E-cadherin

We generated a preliminary protein “homology” model using a mouse E-cadherin crystal structure as template (PDB ID: 3Q2V; resolution = 3.4 Å; sequence identity to human E-cadherin = 82%; coverage = 98%). We used the software Modeller 9.9 after aligning the template with the human E-cadherin sequence (D^155^–I^707^; UniProt ID: P12830) with ClustalW (www.ebi.ac.uk/Tools/msa/clustalw2/)^57^,^58^. The final model was selected by analysing the Ramachandran plots of the computed structures, which we computed with EBI PDBsum Generate (www.ebi.ac.uk/thornton-srv/databases/pdbsum/Generate.html). The best model had 434 (90%) residues in the most favoured regions, with only one problematic residue (V^310^). We did not perform subsequent molecular dynamics relaxation because we only used the model for visual inspection of *H. pylori* HtrA cleavage sites.

## Additional Information

**How to cite this article**: Schmidt, T. P. *et al*. Identification of E-cadherin signature motifs functioning as cleavage sites for *Helicobacter pylori* HtrA. *Sci. Rep.* **6**, 23264; doi: 10.1038/srep23264 (2016).

## Supplementary Material

Supplementary Information
