# Supplementary material for: Identification of E-cadherin signature motifs functioning as cleavage sites for Helicobacter pylori HtrA
Source: Sci Rep. 2016 Mar 17;6:23264. doi: 10.1038/srep23264 (PMC4794652; doi:10.1038/srep23264)
Supplement: Supplementary Information [file srep23264-s1.pdf]

Supplementary information

Identification of E-cadherin signature sites functioning as cleavage sites for *Helicobacter pylori* HtrA

Thomas P. Schmidt[1*], Anna M. Perna[2*], Tim Fugmann[3], Manja Böhm[4], Jan Hiss[2], Sarah Haller[2], Camilla Götz[1], Nicole Tegtmeyer[4], Benjamin Hoy[1], Tilman T. Rau[5,6], Dario Neri[2], Steffen Backert[4], Gisbert Schneider[2#], and Silja Wessler[1#]

Experimental procedures

Cloning and enrichment of hCdh1 deletion mutants. The chimeric construct encoding the extracellular domain (aa 1 – aa 706) of E-cadherin fused to the Fc domain of human IgG is described elsewhere [1]. To delete individual EC domains, *Xho*I restriction sites were inserted in the 5' and 3' flanked regions of EC domains via site directed mutagenesis. After restriction the plasmids were religated, resulting in the deletion of the respective extracellular domains and an insertion of an *Xho*I restriction site (Tab. S1). AGS cells were transfected with Cdh1-Fc constructs and after 2, 3 and 4 days, supernatants containing secreted E-cadherin were collected. The supernatants were cleared by centrifugation at 1000 x g for 10 min and recombinant cadherin-Fc fusion proteins were isolated using sepharose A (GE Healthcare). After incubation with the supernatant, the sepharose was washed and the recombinant E-cadherin was eluted by a pH shift (100 mM glycine-HCl, pH 2.5). The eluate was immediately buffered by addition of 1 M Tris-HCl pH 9.5 and dialyzed in 50 mM Tris (pH 7.5) containing 1 mM $CaCl_2$.

Transepithelial electrical resistance (TEER). To determine TEER of a confluent MKN-28 cell monolayer, the transwell system (Corning) was pre-incubated with cell culture medium for 1 h. Subsequently, MKN-28 cells were cultured on 1.12 cm² cell culture inserts with 3.0 µm pore size (Corning) and incubated for 14 days at 37°C

in 5% CO_2. TEER was measured with an Electrical Resistance System (EVOM) (World Precision Instruments, Inc.) and was calculated as Ohms x cm^2 by subtracting fluid resistance and multiplying by the monolayer surface area.

Table. S1. cDNA constructs used in this study.

**Plasmid**	**Protein**		**Reference**
Cdh1-Fc wt	aa 1 - aa 706 fused to Fc	wildtype	[1]
Cdh1 ΔEC1-Fc	Deletion of aa 155 – aa 262	Deletion of EC1	This study
Cdh1 ΔEC1-2-Fc	Deletion of aa 155 – aa 375	Deletion of EC1-2	This study
Cdh1 ΔEC1-3-Fc	Deletion of aa 155 – aa 486	Deletion of EC1-3	This study
Cdh1 ΔEC4-5-Fc	Deletion of aa 487 – aa 697	Deletion of EC4-5	This study
Cdh1 ΔEC5-Fc	Deletion of aa 594 – aa 697	Deletion of EC5	This study

Figure legends

Figure S1. Specific detection of Cdh1 fragments. (**A**) Scheme of Cdh1-Fc wt and the isogenic EC1, EC1-2, EC1-3, EC4-5, and EC5 deletion mutants. (**B**) Recombinant Cdh1-Fc proteins were separated by SDS PAGE and blotted on membranes. For the detection of the EC1 domain, the membrane was incubated with the SHE78-7 antibody. The EC2 domain was recognized by the HecD1 antibody, and the EC5 domain was detected by the H108 antibody. (**C**) Different cell lines used in this study were colonized by *H. pylori* at a MOI 100 for 16 h. Cells were extensively washed, scraped into medium and plated on agar plates in different dilutions. Colonies were quantified after 72 h growth under microaerophilic conditions. (**D**) Cells were infected with *H. pylori Hp*26695 for 16 h. Aliquots of supernatants were analyzed by Western blot using an anti-EC5 antibody (H108).

Figure S2. HtrA-mediated fragmentation of E-cadherin. (**A**) Long exposure of images shown in Figure 2A to detect additional Cdh1 fragments.

Figure S3. P1 binding to HtrA. (**A**) MS analytics of peptide P1. (**B, C**) SPR sensorgrams for P1 peptide binding to immobilized HtrA. P1 peptide was applied with (**B**) carboxyl (C1-COOH) or (**C**) amide (C1-NH2) C-terminus.

Figure S4. Analysis of the P1 peptide. (**A**) Synthesized peptides based on the most frequent detected peptide during label-free mass spectrometry-based proteomic analysis. Underlined amino acid residues indicate the cadherin consensus sequence. Bold amino acids indicate exchanged amino acids. (**B**) 50 ng recombinant E-cadherin (rCdh1) was incubated with 200 ng recombinant HtrA (rHtrA) for 16 h at 37°C. As indicated, 100 µM of the peptides were added. (**C**) 50 ng rCdh1 was incubated with 200 ng rHtrA for 16 h at 37°C. As indicated, 100 µM or 50 µM of the peptides were included. DMSO was added as a control. The cleavage fragments were analyzed by Western blot and detected using an antibody against the EC5. (**D**) Quantitative analysis of the relative amount of β-actin in whole cell lysates (WCL) of the *H. pylori*-infected cells by blot densitometry from the three independent experiments as shown in Fig. 5B.

Figure S5. Transepithelial resistance (TEER) characteristics of polarized MKN-28 cells using a transwell filter system. MKN-28 cells were grown as a monolayer in a transwell filter system. The cells were then differentiated and TEER was allowed to establish over 14 days as indicated (**A**). MKN-28 cells were incubated over a 24 hour time course in the presence or absence of HtrA inhibitor P1 and infected with *H. pylori* (**B**). TEER measurement was performed as described in the Materials and Methods section.

Figure S6. Original Western blots as shown in Fig. 4B. Analysis of rCdh1 cleavage by HtrA in the presence of P1, P3, P4, P5 (dotted lines) or test compounds t1-t14 (not part of this study). Separate sections of the same membrane are shown in Fig. 4B.

References

1 Niessen, C. M. & Gumbiner, B. M. Cadherin-mediated cell sorting not determined by binding or adhesion specificity. *The Journal of cell biology* **156**, 389-399, doi:10.1083/jcb.200108040 (2002).

Figure S1

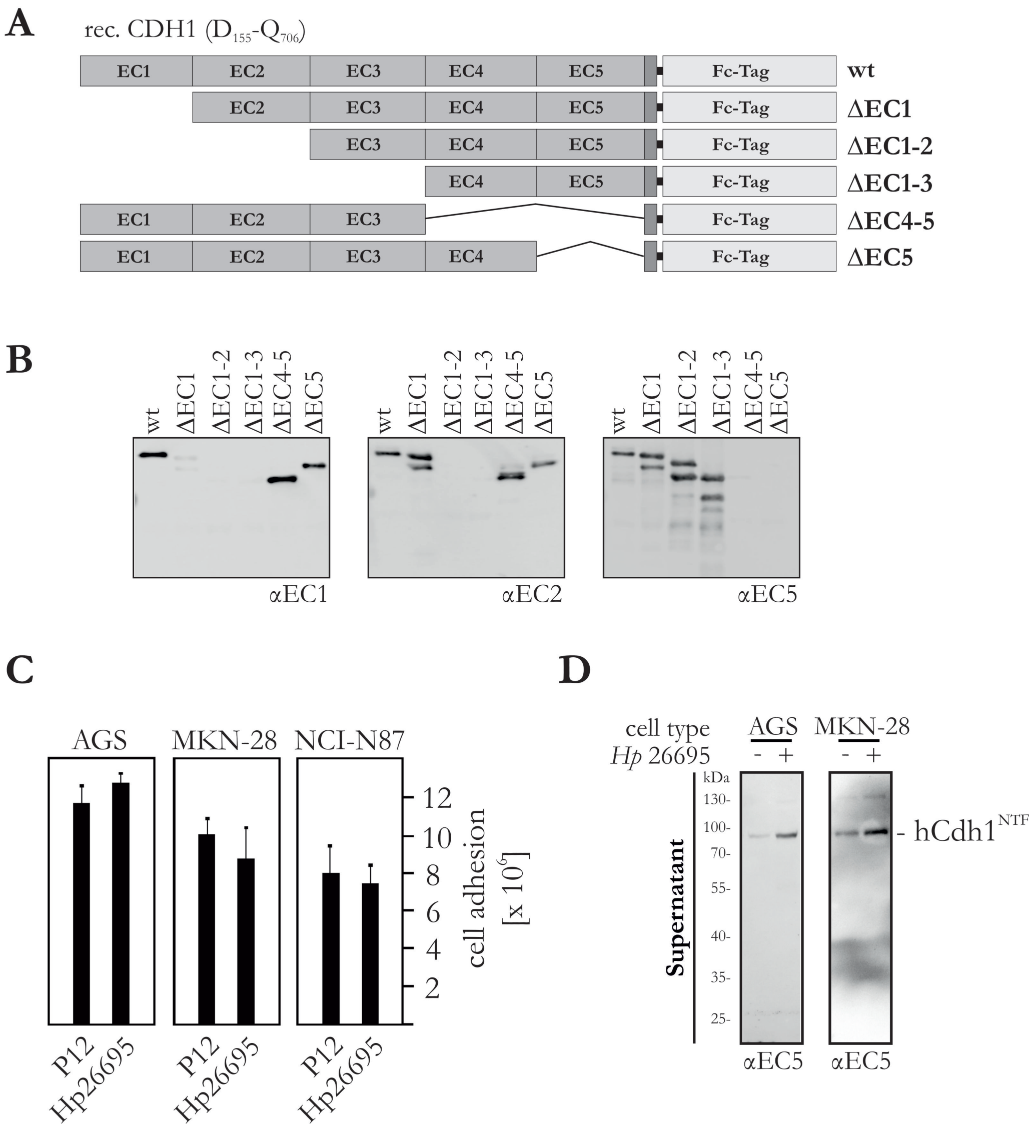
A
rec. CDH1 (D155-Q706)
EC1
EC2
EC3
EC4
EC5
Fc-Tag
wt
ΔEC1
ΔEC1-2
ΔEC1-3
ΔEC4-5
ΔEC5
B
wt
ΔEC1
ΔEC1-2
ΔEC1-3
ΔEC4-5
ΔEC5
αEC1
αEC2
αEC5
C
AGS
MKN-28
NCI-N87
12
10
8
6
4
2
cell adhesion
[x 10^6]
P12
Hp26695
D
cell type
Hp 26695
AGS
MKN-28
-
+
kDa
130-
100-
70-
55-
40-
35-
25-
Supernatant
- hCdh1^NTF
αEC5

Figure S2

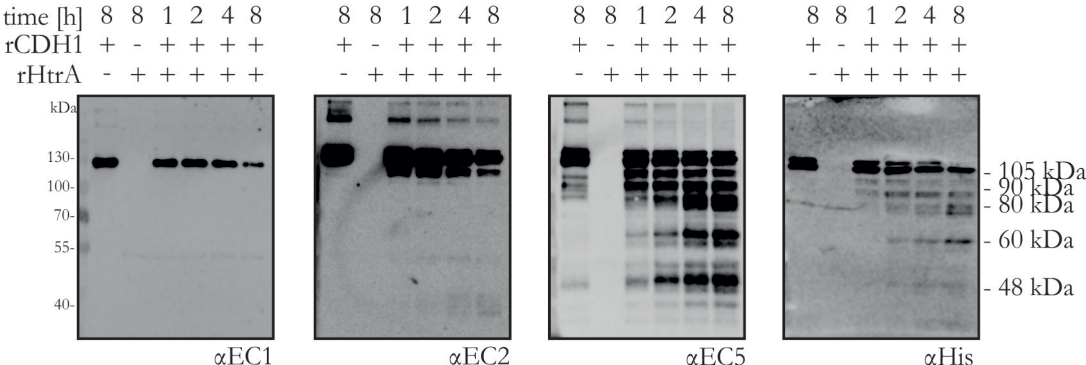
time [h] 8 8 1 2 4 8
rCDH1 + - + + + +
rHtrA - + + + + +
8 8 1 2 4 8
+ - + + + +
- + + + + +
8 8 1 2 4 8
+ - + + + +
- + + + + +
8 8 1 2 4 8
+ - + + + +
- + + + + +
kDa
130-
100-
70-
55-
40-
αEC1
αEC2
αEC5
αHis
- 105 kDa
- 90 kDa
- 80 kDa
- 60 kDa
- 48 kDa

Figure S3

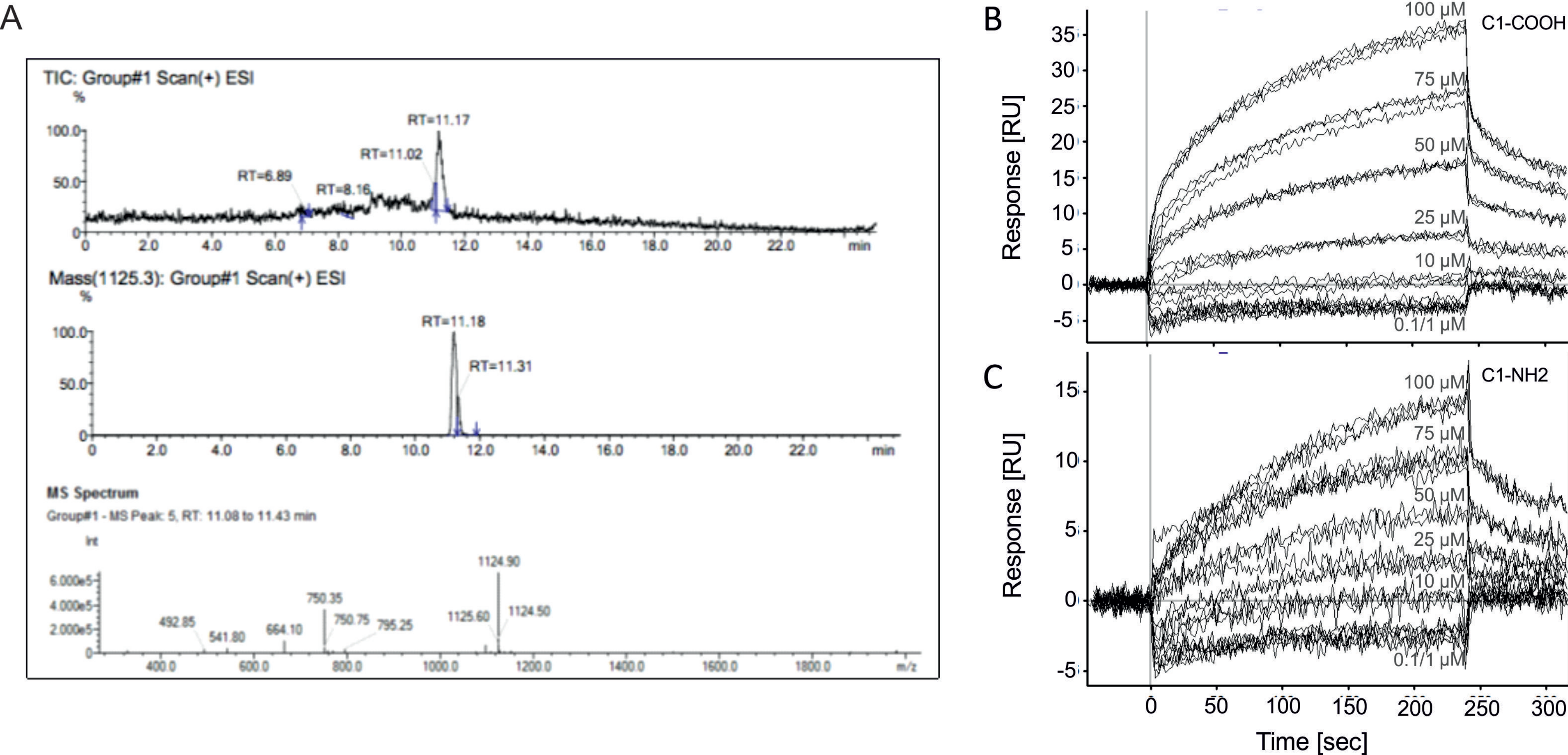
A
TIC: Group#1 Scan(+) ESI
RT=11.17
RT=11.02
RT=6.89
RT=8.16
Mass(1125.3): Group#1 Scan(+) ESI
RT=11.18
RT=11.31
MS Spectrum
Group#1 - MS Peak: 5, RT: 11.08 to 11.43 min
1124.90
750.35
1124.50
1125.60
750.75
795.25
492.85
541.80
664.10
m/z
min
B
C1-COOH
C
C1-NH2
100 µM
75 µM
50 µM
25 µM
10 µM
0.1/1 µM
Response [RU]
Time [sec]

Figure S4

A

**P1**	TGTLLLILSDVNDNAPIPEPR	
**P2**	LLILSDVNDNAPIPEPR	
**P3**	ILSDVNDNAPIPEPR	
**P4**	LSDVNDNAPIPEPR	
**P5**	NDNAPIPEPR	
**P1_NT**	TGTLLLI	
**P1_NT(TG)**	TGT**GTGT**	
**P1_NT(GT)**	**GTGTGTG**	
**P1_CT$_{mut}$**	TGTLLLILS**G**V**GGG**A**G**IPEPR	
**P1_NT$_{mut}$**	TGTL**GGG**LSDVNDNA**G**IPEPR	
**P1$_{mut}$**	TGTL**GGG**LS**G**V**GGG**A**G**IPEPR	synthesis failed

B

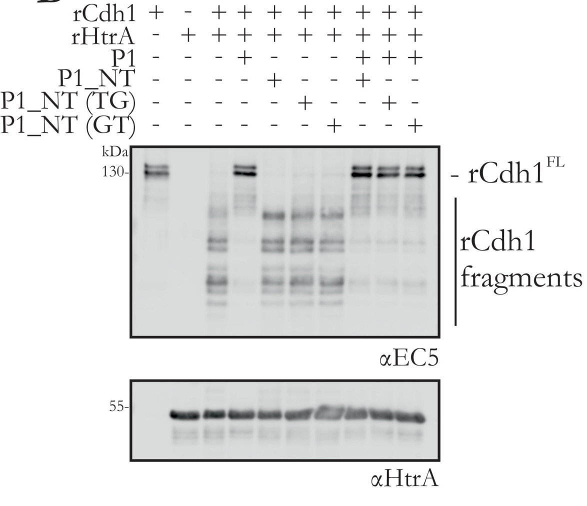

C

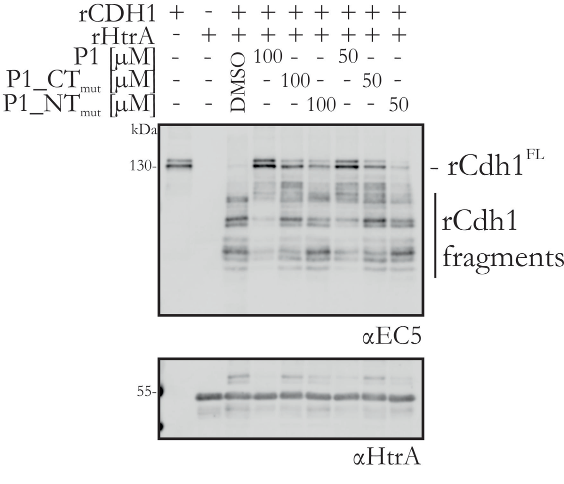

D

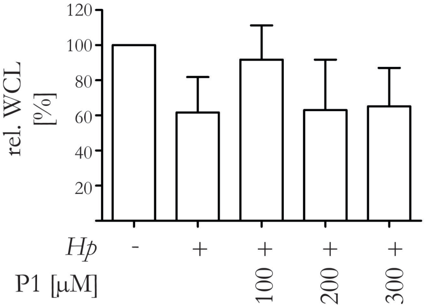

Figure S5

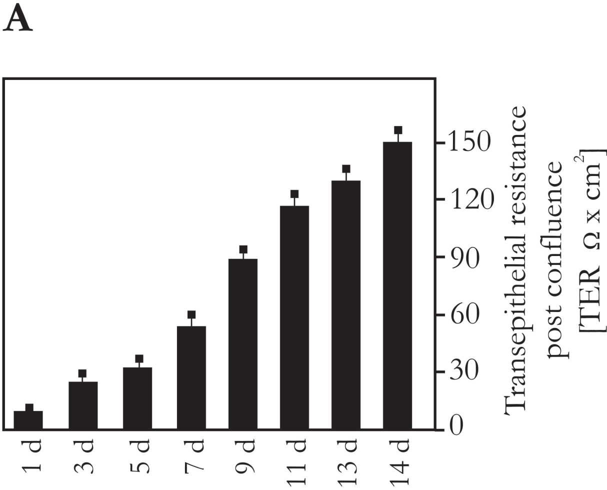
A
Transepithelial resistance post confluence [TER Ω x cm2]
0
30
60
90
120
150
1 d
3 d
5 d
7 d
9 d
11 d
13 d
14 d

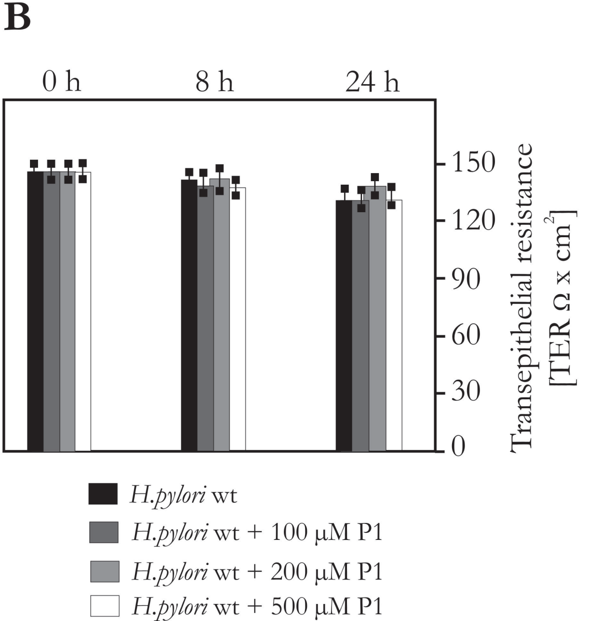
B
0 h
8 h
24 h
Transepithelial resistance [TER Ω x cm2]
0
30
60
90
120
150
H.pylori wt
H.pylori wt + 100 µM P1
H.pylori wt + 200 µM P1
H.pylori wt + 500 µM P1

Figure S6

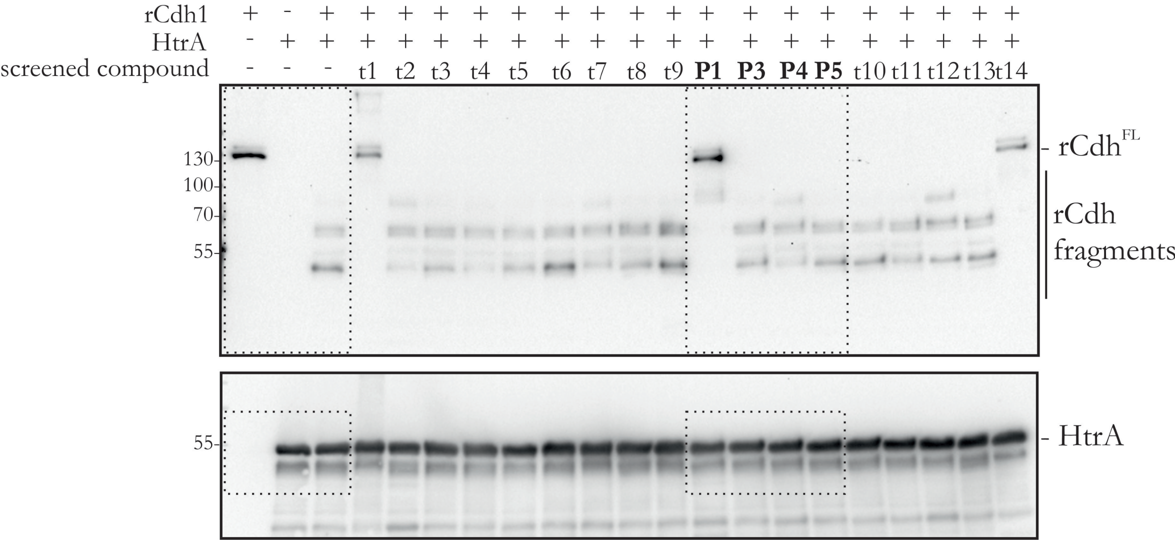

rCdh1 + - + + + + + + + + + + + + + + + + + + +
HtrA - +
screened compound - - - t1 t2 t3 t4 t5 t6 t7 t8 t9 P1 P3 P4 P5 t10 t11 t12 t13 t14
130
100
70
55
rCdh^FL
rCdh fragments
55
HtrA
